# Voices from the field: healthcare professionals’ insights on sustaining telemedicine for diabetes management in Hong Kong primary care

**DOI:** 10.3389/fdgth.2025.1665424

**Published:** 2025-11-28

**Authors:** Arkers Kwan Ching Wong, Luna Ziqi Liu, Frances Kam Yuet Wong, Jun Liang, Danny Wah Kun Tong, Man Li Chan, Man Kin Wong, Bo Chu Wong, Cecilia Yeuk Sze Tang, Wai Hing Ho, Sau Ching Chiang

**Affiliations:** 1 School of Nursing, The Hong Kong Polytechnic University, Kowloon, Hong Kong SAR, China; 2 Hospital Authority, Kowloon, Hong Kong SAR, China

**Keywords:** telemedicine, diabetes mellitus, primary healthcare, NASSS framework, ecological model, implementation science

## Abstract

**Introduction:**

Diabetes mellitus is a prevalent chronic illness that imposes substantial health and financial burdens. In routine follow-up for diabetes, telemedicine offers a promising alternative to traditional face-to-face care within primary care settings, yet real-world adoption remains uneven and often discontinuous. This study explored how healthcare professionals experience the implementation of telemedicine consultations for diabetes management, identifying facilitators, barriers, and resources needed for long-term operation.

**Methods:**

We conducted a qualitative study with 21 healthcare professionals involved in a hybrid telemedicine program in public primary care. Semi-structured interviews underwent a three-stage analysis: first, inductive thematic coding; second, organization of themes using the NASSS framework (Non-Adoption, Abandonment, Scale-Up, Spread, Sustainability); and third, ecological mapping of each NASSS-organized theme to micro, meso, exo, macro, and chrono levels to trace cross-level pathways and temporal shifts.

**Results:**

Thirteen themes were identified and grouped across ecological levels and NASSS domains. Key facilitators included coordinated policy and organizational support, prepared clinic infrastructure, effective training and IT support, and positive perceptions among staff and caregivers. Major barriers included staffing constraints and workflow burden, patient digital literacy challenges and environmental constraints, process complexity including identity verification and e-payment steps, limited suitability for unstable clinical presentations, and gaps in end-to-end service features such as medication delivery.

**Discussion:**

Sustaining telemedicine in primary care will require addressing these barriers while reinforcing enabling conditions through aligned policy and financing, streamlined infrastructure and workflows, targeted patient and staff supports, and continued adaptation over time. The combined NASSS and ecological approach clarifies what the determinants are and where and how they operate, offering level-specific, actionable directions to strengthen the long-term delivery of diabetes care via telemedicine.

**Clinical Trial Registration:**

https://clinicaltrials.gov/ct2/show/NCT05183685, identifier NCT05183685.

## Introduction

Globally, approximately one in three community-dwelling people suffer from one or more chronic illnesses (1), with diabetes mellitus being one of the most prevalent diseases. Diabetes mellitus (DM), which affects at least 10% of Hong Kong's population, is a metabolic disorder characterized by chronic hyperglycaemia due to defects in insulin secretion, insulin action, or both (2). Beyond glycaemic dysregulation, DM drives a broad spectrum of complications. Microvascular sequelae include diabetic retinopathy, nephropathy, and peripheral neuropathy that contribute to vision loss, end-stage kidney disease, neuropathic pain, foot ulceration, and lower-extremity amputation. Macrovascular disease encompasses coronary artery disease, myocardial infarction, stroke, peripheral arterial disease, and heart failure, and remains a leading cause of excess morbidity and premature mortality in people with diabetes (3). The condition also imposes substantial psychosocial and economic burdens on patients, families, and health systems; in Hong Kong, annual healthcare expenditures attribute to DM are approximately US$2 billion (4).

Given the rising prevalence and wide-ranging impacts, primary care units such as General Out-Patient Clinics (GOPCs) in Hong Kong deliver care to millions of patients with chronic illnesses, including diabetes mellitus (DM). The Risk Assessment Management Program (RAMP) provides multidisciplinary risk assessments, complication screenings, and regular follow-up consultations (5). Evidence indicates that these services improve glycaemic control and other cardiometabolic outcomes such as HbA1c and body weight (6–8). However, the reliance on face-to-face visits can limit timely attendance, particularly for patients with mobility constraints or those living in rural areas, where travel to GOPCs is time-consuming and logistically challenging (9). In light of advances in digital health, telemedicine presents a potential alternative that can maintain continuity of DM care while mitigating access barriers.

Telemedicine refers to the remote delivery of clinical services using telecommunications technologies to support assessment, treatment, monitoring, and patient education (10). Numerous studies have demonstrated benefits for diabetes management, including improvements in HbA1c, lipid profiles, and BMI, as well as better adherence to self-care compared with conventional in-person care (11–14). Patients frequently report telemedicine as convenient and empowering because it expands access to care and can positively affect emotional well-being and family routines (15–18).

Despite these advantages, real-world adoption has been uneven, and telemedicine programs launched within research contexts are often discontinued once studies end. This discontinuity underscores the need to understand factors that influence sustained implementation and routine use in practice. In alignment with the Hong Kong Hospital Authority's (HA) strategic vision for telemedicine, we developed the Telemedicine Consultation at GOPC Program (19), building on the existing RAMP model to test a hybrid approach that combines telemedicine with in-person consultations. To inform implementation and sustainability, we conducted a qualitative study among healthcare professionals directly involved in delivering the program. As frontline providers who interface with both the organization and patients, they offer insight into practical workflows, perceived benefits and challenges, and system-level considerations that shape long-term viability.

This study aims to elucidate the experiences of healthcare professionals, identify the facilitators and barriers to sustaining telemedicine, and determine the resources and adaptations necessary for long-term telemedicine services. The insights gained from this study will provide a foundation for future strategies and preparations to integrate telemedicine into routine healthcare practices, thus ensuring its effective and sustainable implementation.

## Method

### Study design

This qualitative study formed part of the process evaluation of the Telemedicine Consultation at GOPC Program, a hybrid telemedicine–in-person care model delivered in Hong Kong's public General Out-Patient Clinics (GOPCs). We used semi-structured, one-on-one interviews with healthcare professionals (*n* = 21) to generate in-depth accounts of barriers, enablers, and contextual dynamics shaping implementation and sustainability. The Standards for Reporting Qualitative Research checklist guided the reporting of the results (20). This study was registered at clinicaltrials.gov (identifier: NCT05183685; https://clinicaltrials.gov/ct2/show/NCT05183685).

### Theoretical framework

**Figure 1:**
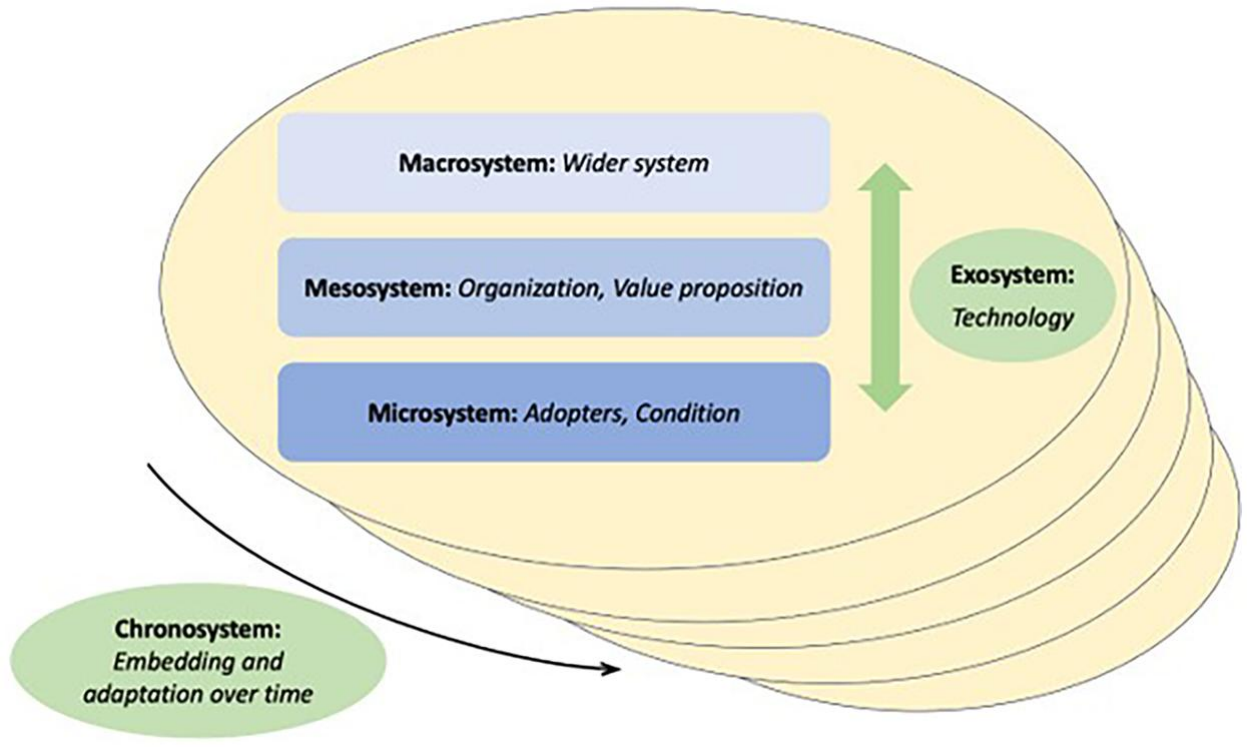
The figure illustrates how the two theories are connected to each other.

To better answer our research question, a combined analytical framework integrating the NASSS framework and Bronfenbrenner's Ecological Systems Theory was applied, offering a multi-layered understanding of both individual and systemic factors that influence the adoption of healthcare technology (Figure 1).

We used the NASSS framework to organize determinants of digital health adoption, non-adoption, scale-up, spread, and sustainability. NASSS specifies seven domains: Condition (the clinical problem and its co-morbidities), Technology (features, dependability, usability, data), Value proposition (benefits and costs for patients, professionals, and the organization), Adopters (patients, caregivers, clinicians and their capabilities and beliefs), Organization (structures, resources, workflows, readiness), Wider system (policy, regulation, reimbursement, market), and Embedding and adaptation over time (how the service evolves) (21–23).

To locate determinants in their broader context, we drew on Bronfenbrenner's ecological model, which distinguishes nested levels of influence: Microsystem (immediate interactions such as clinician–patient encounters), Mesosystem (connections among settings such as coordination between front desk, nursing, and medical staff), Exosystem (indirect structures such as digital infrastructure and vendor arrangements), Macrosystem (societal and policy environment including financing and regulation), and Chronosystem (changes over time in technology, policy, and practice) (24–28).

The following section outlines how these frameworks are integrated.

1. At the macrosystem level, which considers broad societal, cultural, and policy contexts, the Wider System domain from NASSS is classified (24). This level captures how overarching policies, economic conditions, and societal norms influence the scalability and sustainability of telemedicine (21).
2. Within the mesosystem, which focuses on interactions between various environments, the Organization and Value Proposition domains from NASSS are categorized (29). This level examines how the internal practices of healthcare organizations and supply and demand influence its successful adoption (21, 22).
3. At the microsystem level, where direct interactions between individuals are explored, the Adopters and Condition domains from NASSS are classified (29). This allows for an analysis of how personal relationships—such as those between patients, caregivers, and healthcare providers—affect the adoption of telemedicine. Factors like the patient's health condition and the digital literacy of caregivers are crucial in determining adoption at this level (27, 28).
4. The exosystem, representing indirect external influences such as social structures and infrastructures, is aligned with the Technology domain from NASSS (29). This level focuses on external resources that indirectly shape digital health implementation, and also to a certain extent mediate outcomes and behaviors (21).
5. Finally, the chronosystem, which addresses the influence of time and evolving contexts, is aligned with the Embedding and Adaptation Over Time domain in NASSS (25). This system captures how adaptations evolve in response to changes in technology, society, or policy over time (22).

### Rationale for integration and analytic sequence

NASSS was selected as the primary scaffold because it provides domain-specific constructs tailored to health technologies and is widely used in digital health evaluation. We added an ecological lens to specify where each determinant operates (level), how influences at one level shape other levels (cross-level pathways), and when these relationships change (chrono). Although PERCS—adapted from NASSS for remote consultations—is directly relevant to the consultation encounter, our study focused on a system-embedded, hybrid telemedicine model (RAMP + teleconsultation) that required analyzing policy and financing (macro), infrastructure/vendor arrangements (exo), organizational redesign (meso), and their propagation to micro-level encounters over time. We therefore used NASSS to define what the determinant was (domain) and the ecological lens to define where/how/when it operated, while drawing on the PERCS literature in the Discussion to situate our findings.

### Program context (GOPC telemedicine workflow)

The telemedicine program was implemented at six General Out-Patient Clinics (GOPCs) located in the New Territories West Cluster in Hong Kong. These GOPCs provide comprehensive primary care services tailored to the needs of patients with chronic diseases and collectively serve more than 50,000 diabetic patients per year (30). Their services include regular follow-ups, medication management, health education, and lifestyle counseling. Both the intervention and control groups received the RAMP service facilitated by designated doctors and nurses at the GOPCs. A full description of the study protocol is available elsewhere (19).

The telemedicine service for the intervention group was provided through the “HA GO” app, an application designed by the Hong Kong Hospital Authority to integrate healthcare services with electronic health records and medical history systems. A newly added feature named “Telemedicine” and an online consultation function were integrated into HA GO, allowing consultations to be conducted in a virtual meeting room directly within the app. The process of using the telemedicine service mirrored those of traditional face-to-face consultations. At the end of each consultation, doctors would schedule the next appointment and upload the details to the medical system, which were then linked to the HA GO app. On the day of the appointment, patients first checked their appointment information in the HA GO app, and then completed payment using the built-in e-payment feature. Thirty minutes before the consultation, a clerk from the GOPC would call the patient to provide reminders and assist with pre-consultation procedures, including verifying their identity. At the scheduled time, the clerk would assist the doctor and the patient in joining the virtual meeting room to conduct the consultation. Depending on the patient's condition, the online consultation could accordingly be transferred to a nurse clinic. If medication was required, the patient or an authorized person had to collect the prescription from the GOPC's pharmacy within four days of the doctor's writing of the prescription.

### Setting and participant recruitment

Healthcare professionals who were responsible for delivering the 84-week RAMP service for the intervention group in the Telemedicine Consultation at GOPC Program were recruited for this qualitative study. Those professionals all possessed extensive experience in both traditional face-to-face consultations and telemedicine. This ensured that the study captured a wide range of insights into the effectiveness of the telemedicine program and areas for improvement. Purposive and convenience sampling strategies were employed to ensure a diverse and representative sample, allowing for the exploration of various perspectives and experiences related to the telemedicine intervention (31).

### Data collection and procedure

A total of 21 one-on-one interviews, each lasting approximately 40–60 min, were conducted with the participants, who included advanced practice nurses (APN), ward managers (WM), doctors (D), and clerks (C) from six GOPCs. These qualitative, semi-structured interviews took place face-to-face in a private room within the clinics, ensuring a conducive environment for detailed and candid discussions. The interviews were completed within a month after the intervention ended to ensure that the participants had a fresh and deep recollection of their experiences with the telemedicine program. The interviews were conducted by three research team members with experience and advanced training in qualitative research.

A semi-structured interview guide was applied. All of the interviews were digitally audio-recorded and transcribed, and the transcripts were de-identified to maintain participant confidentiality. The de-identified transcripts were then translated into English for data analysis. The transcription and translation were conducted by team members who were proficient in both Cantonese and English, ensuring accuracy in capturing the nuances of the participants' responses. The translated and de-identified transcripts were imported into NVivo 14 for data analysis.

### Data analysis

We used a three-stage analytic sequence: (1) inductive thematic coding, (2) organization of themes using NASSS domains, and (3) ecological mapping of each NASSS-organized theme to micro, meso, exo, macro, and chrono levels, including tracing cross-level pathways (for example, policy guidance → clinic scheduling rules → eligibility decisions → patient experience) and temporal shifts.

Stage 1: Inductive thematic coding. We conducted a thematic analysis to develop codes, subthemes, and themes directly from the interview transcripts. Two researchers repeatedly read the transcripts to gain an in-depth understanding of participants' experiences and perspectives. Open coding was independently performed on four transcripts to identify meaningful segments relevant to the research aims, producing an initial coding frame. Through constant comparison and team discussion, this frame was iteratively refined and then applied to all transcripts. Initial codes were grouped into broader categories and then into themes that described key facilitators and barriers to implementing and sustaining telemedicine. Themes and codes were reviewed iteratively to ensure faithful representation of the data, with disagreements resolved by discussion and consensus, and the process documented in an audit trail (32).

Stage 2: NASSS domain organization. Next, we used the NASSS framework to structure the thematic output. All codes and themes were systematically assigned to NASSS domains (Condition, Adopters, Technology, Value Proposition, Organization, Wider System, Embedding and Adaptation Over Time). We documented decision rules for domain assignment, reviewed the mapping in team meetings, and resolved any discrepancies through discussion to enhance transparency and reproducibility.

Stage 3: Ecological mapping and cross-level interpretation. Finally, we applied an ecological systems lens to locate each NASSS-organized theme at the appropriate level of influence (microsystem, mesosystem, exosystem, macrosystem, chronosystem). We constructed matrices that linked domains to levels and explicitly traced cross-level pathways and time-related adaptations. This step moved beyond domain listing by explaining how determinants interacted across levels and evolved over time. The initial classifications and interpretations were reviewed by the full team to ensure accuracy, and were further validated through cross-referencing with existing literature and expert discussions (33).

### Ethical considerations and methodological trustworthiness

The study was conducted under the standards and ethical criteria of the Helsinki declaration and approved by the ethics committee of the Hong Kong Polytechnic University (HSEARS20200619003). To ensure trustworthiness, Lincoln and Guba's framework for establishing rigor was applied (34), encompassing credibility, confirmability, transferability, and dependability. Credibility was achieved by having trained researchers fluent in Cantonese and English conduct all of the interviews and processing all of the data. This facilitated an in-depth exploration of the participants’ experiences. Confirmability was ensured by maintaining an audit trail and using reflexivity to minimize researcher bias, documenting all decisions made during the research process. Transferability was achieved through detailed descriptions of the study process, context, and participants, using thick descriptions to allow others to assess the applicability of the findings to different settings. Dependability was ensured by consistent documentation and by maintaining an audit trail throughout the research process, with regular methodological reviews to ensure rigor. This approach ensured the validity and reliability of the findings.

## Results

### Socio-demographic characteristics

A total of 21 participants, made up of various healthcare professionals, were included in this study. The sample included 5 nurses, 3 ward managers, 8 doctors, and 5 clerks. The participants ranged in age from 20 to over 60 years and included 14 females and 7 males. The participants' experience in the GOPC ranged from 3 to over 20 years, and their experience with telemedicine ranged from 1 to 5 years. They were therefore a well-rounded sample, with substantial expertise in both traditional and telemedicine settings. Further details about the demographic characteristics of the participants are provided in Table 1.

**Table 1:** Demographic data of the participants.

Healthcare Professionals	Gender	Age Range	Clinic	Work Title	Exp in GOPC (years)	Exp in telemedicine (years)
APN01	F	50–59	Clinic A	Advanced practice nurse	20	2
APN02	F	50–59	Clinic B	Advanced practice nurse	20	4
APN03	F	50–59	Clinic E	Advanced practice nurse	20	3
APN04	F	50–59	Clinic D	Advanced practice nurse	10	5
APN05	F	30–39	Clinic F	Advanced practice nurse	11	1
C01	F	30–39	Clinic A	Clerk II	9	3
C02	M	20–29	Clinic B	Executive Assistant III	5	2
C03	M	20–29	Clinic E	Executive Assistant II	1.5	1
C04	F	40–49	Clinic D	Clerk III	8	5
C05	F	60+	Clinic F	Executive Assistant II	12	2
D01	F	40–49	Clinic B	Associate Consultant	20	2
D02	M	30–39	Clinic C	Resident Specialist	5	1
D03	M	30–39	Clinic D	Medical Officer	5	1
D04	M	20–29	Clinic D	Medical Officer	4	2
D05	F	30–39	Clinic A	Medical Officer	5	2
D06	M	50–59	Clinic E	Service Resident	10	1
D07	F	50–59	Clinic E	Senior Medical Officer	20	2
D08	M	40–49	Clinic A	Associate Consultant	19	3
WM01	F	40–49	Clinic B	Ward Manager	19	2
WM02	F	50–59	Clinic A	Ward Manager	20	2
WM03	F	40–49	Clinic D	Ward Manager	20	2

### Analyzing the results

To foreground the frameworks' relative contributions, we indicate for each theme whether the primary insight was domain-specific (NASSS) or cross-level/temporal (Ecological); themes marked “Both” reflect domain identification via NASSS with added explanation of how determinants interacted across levels (ecological lens). A total of 13 themes emerged as the main influencing factors. They were categorized according to our analytical framework, followed by 24 codes that acted as specific facilitators or barriers (Table 2).

**Table 2:** Influencing factors.

Primary analytic lens	Ecological	NASSS domain	Theme	Facilitators & barriers
Ecological	Macrosystem	Wider system	Synergizing societal stakeholders and resources	+ Multifaceted support from multiple stakeholders + Proactive promotion
Both	Mesosystem	Organization	Clinics’ environment and technical resources	+ Well-prepared setting and facilities in the clinic
			Managerial and procedural considerations in clinic	+ Adjustments and arrangements within the clinic − Insufficient manpower and overwhelming workload
Both		Value proposition	User experience and feedback	+ Convenience brought about by telemedicine − Lack of incentives and benefits
NASSS	Microsystem	Adopters	Adopters’ perceptions and attitudes	+ (Staff) positive perceptions − (Patients) negative perceptions and concerns arising from telemedicine − (Patients) reluctance to adopt and embrace telemedicine as a viable alternative to in-person visits
NASSS → Ecological (Cross-Level barriers)			Patients’ technological competence and digital literacy	− Lack of knowledge and skills necessary for telemedicine
NASSS			Patients’ environment and technical resources	− Unsatisfactory environment and underdeveloped equipment
NASSS			Caregivers’ involvement	+ Caregivers’ support in technology
NASSS		Condition	Suitability of telemedicine for diabetic patients	− Unsuitable for unstable and complicated cases
Both	Exosystem	Technology	Technical training and support	+ Training and guidance for patients + Technical training and support on IT-related issues for HCPS
Nasss → Ecological (workflow chain)			Technological elements	+ Well-designed app and its features − Challenges posed by e-payment tools − Intricate steps and lack of smooth linkages between telemedicine process steps − Unable to provide physical examinations and face-to-face communication
Ecological (time)	Chronosystem	Embedding and adaptation over time	Experiences of Implementing Telemedicine during the COVID-19 Pandemic	+ Finished guidance and training for HCPs on telemedicine + Patients’ former experience in using telemedicine during Covid + Prepared setting and facilities since Covid
Ecological (policy-to-practice)			Additional supporting services and policies	+ Recommendation for Implementing Medication Delivery

“NASSS” = primarily domain identification; “Ecological” = primarily cross-level/chrono explanation; “Both” = NASSS determinant with ecological interdependencies/temporal dynamics made explicit.

### Wider system (macrosystem)

#### Synergizing societal stakeholders and resources

Wider systems relate to broad societal, cultural, and policy contexts, which significantly influence programs by affecting their ability to transition from successful demonstration projects to fully mainstreamed, scalable, and sustainable services (21). Synergizing societal stakeholders and resources from a comprehensive, overarching perspective serves as a facilitator for sustaining programs. HCPs often noted that certain efforts could not be accomplished by individuals or single organizations alone, emphasizing the need for systemic coordination and macro-level resource allocation to provide adequate support.

Multifaceted support from stakeholders, guided by a unified policy blueprint, including government entities, the Hospital Authority, community groups, and social media influence, ensured a comprehensive network of assistance. The government and Hospital Authority were expected to provide macro-level policy support to promote telemedicine. Community support involved collaborations with clinics to organize training sessions on telemedicine, so as to improve efficiency and bridge technological gaps between individual capabilities and organizational responsibilities. Such comprehensive support was crucial for the long-term sustainability of telemedicine services.

Additionally, in recent years, within the HA, the development of telemedicine has been emphasized in the five-year plan. Therefore, I believe it is important for us to move forward in line with this direction. In the private sector, telemedicine initiatives are already being implemented extensively. I believe that the HA should also adopt such measures, as they facilitate patient care. (D02)

Proactive promotion, combined with efforts to bridge the information gap, significantly increased awareness and acceptance of telemedicine among patients and healthcare providers. Multiple stakeholders employed diverse strategies, such as creating posters and brochures in clinics and community centers, as well as integrating relevant information into television programs. These initiatives provided the public with accessible channels to understand and become familiar with this new approach, highlighting its advantages.

For promotion, perhaps considering advertising on television would help. Although people have heard about video consultations during the pandemic, they may think it's only temporary. So it might be necessary to seek government assistance and promote it on television to provide more information to the public. (C04)

### Organization (mesosystem)

#### The environment and technical resources of clinics

Clinics, as the primary units for providing remote services, rely heavily on their facilities, service capabilities, and internal management to determine the success of the implementation of telemedicine. The infrastructure and technological environment established during the pandemic laid a solid foundation for the current service. Well-prepared settings and facilities, including stable network connections and necessary devices like iPads and cameras, played a crucial role in supporting telemedicine services.

Actually, when I started doing tele from the designated clinic for Covid, we already had a setup in place. We have been using that setup and just made some adjustments to adapt it to the current setup. (APN01)

#### Managerial and procedural considerations in clinics

Healthcare professionals reached the consensus that management and procedural adjustments at the organizational level are required for the successful implementation of telemedicine. Facilitating factors are as follows. Designated meetings for telemedicine personnel, especially in the early stages of implementation, facilitated timely communication and adjustments to service details and models. Where feasible, assigning additional or dedicated personnel for telemedicine was important, as telemedicine increased workloads, particularly for clerical staff responsible for connecting with patients and verifying identities. Establishing dedicated sessions or timeslots for telemedicine allowed HCPs to focus more effectively on their primary responsibilities without frequently switching modes during a fixed timeframe, thereby saving adaptation time. Implementing standardized working regulations and workflows was helpful in providing guidance for HCPs, ensuring optimal efficiency control and effective personnel management.

I think that the higher management should have monthly meetings involving doctors and nurses, at least these two crucial parties…. This provides an opportunity for communication regarding operational matters. As frontline staff, we can share our opinions, like feedback on the current mode with our clinic In-charge…. Also, I would prefer to have a dedicated section where colleagues are assigned to handle telemedicine exclusively. We can concentrate suitable patients in one section, and the assigned colleague can handle all the tele cases in that section. This would make it more convenient. (D02)

However, barriers such as insufficient manpower and excessive workloads negatively affected both the motivation of HCPs and the effectiveness of the implementation of telemedicine. Introducing telemedicine led to reports of staffing shortages and overwhelming workloads across the clinic. The burden fell most heavily on clerical staff, who had to spend extra time managing logistics, building connections between patients and the clinic, assisting doctors and nurses in setting up virtual consultations, performing identity verification, and explaining telemedicine services to patients. These additional responsibilities severely impacted their daily reception tasks. Management professionals noted that the current resources of clinics were already stretched thin, making it difficult to effectively support the expanded implementation of telemedicine.

However, in the case of video consultations, the clerks have additional responsibilities. They need to contact patients, do registrations, when they will pick up the medicine. And after the video consultation, they have a stack of documents that they need to come back and retrieve. They need to help record when the patient will come to pick up the documents and ensure that they have everything. This is for safety and to ensure the entire cycle. From the moment that they are registered, to when they see the doctor, and until they come back to collect the medications, everything needs to be done to ensure that there are no omissions. I think the clerks have a heavier workload; especially when we can't handle things ourselves, we have to rely on them. They have to do many different things and support us technically. This matter is related to manpower. (APN04)

### Value proposition (mesosystem)

#### User experience and feedback

Value assessment pertains to the worth of developing and sustaining new technology, integrating insights from both individual and organizational experiences to inform decision-making processes (21). User experience and feedback serve as critical demand-side considerations that directly influence the adoption and sustainability of the program.

The convenience that telemedicine brought to patients was highly valued, particularly for reducing the need to commute and providing easier access to healthcare services. When patients experienced the ease and accessibility of telemedicine, they were more inclined to accept and continue using remote services. This especially benefited patients with mobility issues, those living in remote areas, and working individuals who did not have enough time to visit clinics in person.

Once they overcome those challenges, they find the service convenient and appreciate the flexibility it offers in terms of time and traffic. It's important that they are willing to keep participating. (D01)

Correspondingly, the lack of incentives and perceived benefits was primarily associated with specific demographic characteristics, such as occupation, age, and IT literacy. The perceived attractiveness of online consultations, in terms of convenience and time-savings, was less compelling for non-working individuals or elderly patients, who often had more time and thus did not value the time-saving aspect as much as others. Moreover, they might have faced challenges and resistance to acquiring IT skills and setting up the required technology, which made it difficult or unappealing for them to utilize telemedicine.

They didn't see any benefits and thought that installing HA GO was pointless. They would say things like, “I don’t need to install HA GO, I can just go to see the doctor and get my medicine myself.” They couldn't see the advantages, so they wouldn't apply it. (WM01)

### Adopters (microsystem)

#### Perceptions and attitudes of adopters

The adopter system, as the smallest unit in healthcare delivery, includes healthcare staff, patients, and caregivers, whose attitudes, abilities, and engagement significantly influence the pace of the implementation of technology.

The attitudes and perceptions of healthcare staff were generally positive and supportive, and were primarily based on their professional recognition of the effectiveness and safety of telemedicine services. Additionally, some healthcare professionals, with years of experience and an understanding of international practices, recognized the potential and advantages of telemedicine. They held a forward-looking perspective that caused them to identify telemedicine, smart healthcare, and virtual care as emerging trends. This contributed to their positive outlook. Their vision and role as pioneers acted as catalysts for advancing telemedicine.

We certainly aim to sustain it, and I believe the goal is not just to maintain but to further develop it. I feel that the direction mentioned earlier—digitalizing everything, especially in managing medication delivery—will help ensure that sustainability and development. (D08)

In contrast, the negative perceptions and concerns of patients acted as significant barriers, often leading to reluctance and resistance to adopting telemedicine. Some patients found telemedicine cumbersome, particularly the setting up of the application, the login process, and the need to return to the clinic for medication, which they perceived as inconvenient. Additionally, some patients were concerned about scams and the risks associated with online consultations, as virtual consultations required online identity verification, the uploading of relevant documents, and the making of online payments. During recruitment, many patients exhibited a strong preference for in-person consultations and showed resistance to telemedicine. Even after participating, some patients dropped out without providing notable reasons.

They often express concerns about fraud and choose not to answer calls, especially when there is no caller ID or if the displayed phone number is unfamiliar to them. If they don't answer, we can't guide them in using telemedicine. (APN02)

#### Technological competence and digital literacy of patients

A lack of the knowledge and skills necessary to make use of telemedicine emerged as a significant barrier on the patients' end. Navigating telemedicine platforms was particularly challenging for patients with low IT literacy, especially elderly individuals, leading to frustration and reduced engagement. Memory issues were also a problem. Although the clinic's staff informed patients about the specific use of telemedicine when booking appointments, many patients often forgot these instructions by the time of their next follow-up consultation. Since virtual consultations did not require prior travel arrangements, some patients even forgot their scheduled appointments.

After recruiting them, it's easy to explain things briefly, but once they go back home and come for a follow-up appointment after a few months, they often forget how to use it…. It's challenging for them; even if we explain it well to them now, they might forget when they return home. They might not remember how to log in or that it's not an in-person appointment but a remote one over the phone. (APN03)

#### Environment and technical resources of patients

An unsatisfactory environment and poor equipment for conducting telemedicine posed significant challenges for patients. Some individuals held online consultations in public places, such as while on public transportation or in an office. This led to privacy risks, particularly while their identity was being verified and discussions were being held of their medical conditions. Additionally, many patients experienced poor internet connections, hindering their ability to engage effectively in online consultations.

I have encountered cases where they had poor Wi-Fi reception, and they couldn't establish a stable internet connection. After experiencing difficulties, they find it troublesome and may decide not to engage in telemedicine in the future. (APN02)

#### Involvement of caregivers

The involvement of lay caregivers played a crucial role in facilitating online services, particularly for elderly individuals who were unfamiliar with the technology. Family support, especially from the younger generation who assisted with the IT setup and with navigating the telemedicine applications, significantly enhanced the experience of the consultation for older patients. In some cases, the caregiver not only addressed the practical challenges of using telemedicine but also provided emotional support, making the process more manageable and reliable, and encouraging sustained use.

Usually, if there are family members available at home and they have the electronic payment methods on their own mobile phones, they can help the patient make the payment in advance. This allows the patient to directly log into their account and proceed with the video consultation, making the whole process smoother. (C02)

### Condition (microsystem)

#### Suitability for diabetic patients

The Condition is the core issue that needs to be addressed, and a critical risk factor, requiring key consideration to determine which potential end users are “suitable” for the technology (21). Telemedicine posed adoption barriers for diabetic patients with poorly controlled conditions or complications, as their unstable health status increased the risk of deterioration and required detailed physical assessments—which is a deficiency of remote services.

For the patients, I feel that if it’s a very stable follow-up case, I believe the patient would be happy to continue using telemedicine. However, if they start developing complications, for example, diabetes can lead to kidney problems, swollen legs, shortness of breath and such, then relatively speaking, in-person consultations would be a bit better as physical examinations can be done. That is, the appearance of complications would hinder their continued use of telemedicine as there are concerns about deterioration. (D05)

### Technology (exosystem)

#### Technical training and support

Beyond the primary entities mentioned earlier, technology, along with predictable advancements and improvements in required knowledge, serves as an external moderating mechanism that influences the implementation, effectiveness, and sustainability of the program (21). The enabling role of technical training and support for both patients and staff was highlighted. For patients, training needed to include an introduction to telemedicine, detailing accessible services and existing limitations. It needed to cover the conditions under which telemedicine could be used, and to offer comprehensive instructions on navigating telemedicine services, including the appointment and consultation processes and the use of relevant apps.

Maybe if we explain it to them again the day before, it might be better. But this would require someone to call and teach them. And then explain it in more detail, telling them that it might be better that way. (C05)

For healthcare professionals, comprehensive training supplemented by IT professional support was found to be more effective than relying on self-directed learning. Compared to patients, staff needed to become proficient in managing various settings and systems across different devices, making the required training more up-to-date, targeted, and comprehensive. Additionally, backup support from dedicated IT professionals was considered both effective and essential when special circumstances arose.

I feel that IT support is important for our staff…. We discovered those issues after experiencing many mistakes over the past year. Not all of our administrative staff are familiar with IT-related matters. Some might be knowledgeable about smartphones, but they might be more familiar with using Apple devices while others prefer Android. When they have to explain these things to different patients, they will encounter situations they are unfamiliar with. (APN05)

#### Technological elements

A well-designed app and its features significantly enhanced usability and improved patient-provider interactions. Designed by the hospital authority, HA GO connected to electronic medical records for online registration and appointment checks. The built-in virtual meeting room feature further facilitated seamless online consultations within the same app. The stability and reliability of the technical infrastructure instilled confidence in both patients and healthcare providers, enhancing their willingness to make use of telemedicine services.

However, an unexpected barrier emerged regarding e-payment tools. Online consultations required patients to complete payments online before accessing the consultation interface, inadvertently creating an additional challenge and higher technical demands, particularly for those unfamiliar with digital payment systems. Furthermore, some patients’ resistance to e-payments, largely attributed to concerns about online scams, ultimately weakened their willingness to engage with telemedicine.

When they hear about so many different types of e-payments, they have some resistance…. The feedback they gave me was that as soon as they entered the e-payment area, they didn't know how to operate it. So, they couldn't handle the e-payment, and as a result, they couldn't do anything else afterwards. (APN01)

Another barrier lay in the app usage process, which featured intricate steps and lacked smooth transitions between stages, making the user experience cumbersome and less efficient than it could have been. Under the current settings and usage protocols, identity verification was required at least three times for each consultation. Patients needed to verify their identity during the app account login, before entering the virtual meeting room, and during meetings with clinic staff, by reporting their ID number or showing their identification on video. This led to unnecessary waiting times and feelings of mistrust, frustration, and impatience among patients, ultimately heightening resistance to telemedicine.

Additionally, the lack of smooth transitions between steps in the process—such as the handover from the virtual waiting room to doctors and nurses—combined with patients leaving or becoming unavailable at the scheduled time, resulted in prolonged waiting periods. Unlike in-person visits, where patients and healthcare providers are physically present in the clinic and can see the queue progress, online consultations required both parties to wait around the estimated time without any prior notice. This inefficiency disrupted normal workflows within the clinic, diminishing the enthusiasm of healthcare providers for using telemedicine.

But we still need to go through some procedures, like verifying the patient's identity, for example, asking them to show their ID card. When patients are waving the ID card around, we can't see it clearly, so we have to tell them to hold it still. Those things do take time. (D07)

Furthermore, the inability to provide physical examinations and the lack of face-to-face communication remain inherent limitations of telemedicine. Some patients believed that the absence of physical examinations compromised the quality of diagnosis and treatment, leading to increased skepticism about the effectiveness of telemedicine. Meanwhile, some patients frequently complained that online consultations were impersonal and lacked warmth, which contributed to their anxiety and reluctance to engage in telemedicine.

There are also some patients who feel that their condition is unstable, or some patients who are very concerned about their physical condition or changes to it, and they would like to have a physical check-up every time…. They may feel, “I need to come back to the clinic. Via tele, you don’t have my blood pressure measured, and you don’t see me, and the doctor doesn’t do a check-up for me.” (APN05)

### Embedding and adaptation over time (chronosystem)

#### Experiences of telemedicine implementation during COVID-19

Embedding and adaptation over time reflect how adjustments evolve in response to technological advancements, societal shifts, or policy changes (22). The experience gained during the pandemic provided valuable insights and resources for integrating telemedicine into routine healthcare services, offering a solid foundation in terms of service delivery and infrastructure readiness in clinical settings.

During the pandemic, healthcare professionals gained extensive experience in utilizing virtual services. Comprehensive training and hands-on practice ensured that staff were well-prepared to conduct virtual consultations effectively during the implementation phase. Additionally, the prior exposure of patients to telemedicine and their positive experiences with virtual healthcare during the pandemic made for a smoother transition to the new approach. To support remote services, clinics also equipped themselves with appropriate electronic devices and reinforced network stability, laying the foundation for current remote service capabilities.

The experience most people had during Covid has accelerated the popularization of this whole thing. (D08)

#### Additional supporting services and policy

Recommendations for implementing the delivery of medications served as a catalyst to accelerate and promote the adoption of telemedicine. Under current policies, patients who participated in teleconsultations still had to physically visit the clinic to obtain prescriptions, which hindered the completeness of telemedicine. Providing medication delivery services following telemedicine sessions would significantly enhance service quality and fully realize the potential benefits of telemedicine.

To make it smoother, medicine delivery services would help. Some patients ask if they can receive their prescribed medications through video consultations. We have informed them that we are gradually implementing medicine delivery services. If we can implement it soon, I believe remote medical services will become more popular. (C04)

## Discussion

### Main findings

This study explores the multi-layered facilitators and barriers impacting the sustainability of telemedicine for diabetes management. Utilizing an ecological systems perspective alongside the NASSS framework, the findings are structured across five ecological levels—macrosystem, mesosystem, microsystem, exosystem, and chronosystem.

At the macrosystem level, policy and societal support emerged as foundational elements driving telemedicine's sustainability. Coordinated support from governmental agencies, healthcare authorities, and community organizations, along with policy frameworks that provide clear collaborating structures and facilitate technology access, is crucial for raising public awareness and promoting the growth of telemedicine (35).

At the mesosystem level, organizational processes and resource availability within clinical settings directly influenced the practical implementation of telemedicine. Clinics with established telemedicine protocols and adequate technical resources had smoother telemedicine experiences, in line with studies that showed the importance of organizational readiness for successful adoption (36). However, issues caused by implementing new services, such as understaffing and high workloads, led to organizational strains that significantly impacted the sustainability of telemedicine (37, 38). This, in turn, highlights the importance of the effective allocation of organizational resources, along with timely communication and adjustments through phase reviews, to maintain operational efficiency and support sustainability.

The microsystem, which represents direct interactions between patients and healthcare providers, played a significant role in shaping the acceptance and applicability of telemedicine. Overall, healthcare providers and caregivers contributed positively, in terms of both their attitudes and competencies. However, the primary barriers were concentrated on the side of the patients, with digital literacy, availability of resources, and comfort with digital platforms—particularly among older adults—being critical for sustained telemedicine use (36). Furthermore, telemedicine was often unsuitable for patients with unstable or complex conditions due to the inability to perform physical assessments, which limited its application and underscored the essential role that individual skills and health conditions play in the adoption of technology (27, 39).

At the exosystem level, which captures the indirect influence of technological infrastructure and support, both comprehensive training and robust IT support emerged as facilitators of telemedicine sustainability. This demonstrated that integrated technical support systems significantly enhance the usability and reliability of telemedicine across patient populations (36). However, barriers such as a complex identity verification system and e-payment challenges posed obstacles for less tech-savvy patients. This finding is consistent with that of prior research indicating that simplified, user-friendly technological systems are essential for fostering patient engagement in telemedicine (36, 37).

Lastly, the chronosystem reflects the adoption of telemedicine over time, a process that was accelerated by the COVID-19 pandemic. The pandemic-driven experiences provided both patients and providers with prolonged exposure to telemedicine, fostering greater familiarity and acceptance of the technology (39, 40). Additionally, the future integration of services such as medication delivery post-consultation promises to enhance the comprehensiveness and convenience of telemedicine for chronic disease management. Studies indicate that these add-on services not only boost patient satisfaction but also significantly improve adherence and health outcomes, underscoring the importance of supporting continuity of care (38, 41).

Our findings align with prior NASSS-informed and PERCS-based studies of remote consultations in identifying organizational readiness, workflow redesign, and digital inclusion as pivotal determinants of telemedicine uptake and sustainment. PERCS, developed from NASSS to evaluate remote consultations, emphasizes consultation-level processes and roles; our results corroborate these insights while extending them in a system-embedded, hybrid primary-care model. Specifically, by combining NASSS with an ecological lens, we delineate cross-level mechanisms—for example, policy guidance (macro) → scheduling and manpower rules (meso) → eligibility decisions and identity verification steps (micro) → patient experience and continuity, and we trace time-dependent adaptations (chrono) as COVID-era practices persisted or receded. This cross-level, temporal mapping yields level-targeted recommendations (macro reimbursement alignment, exo infrastructure/vendor simplification, meso workflow standardization and staffing, micro training and caregiver supports) that complement the consultation-focused insights typically emphasized in PERCS studies.

### Integrating telemedicine into local primary healthcare for diabetes management

Integrating telemedicine into local primary healthcare settings presents significant opportunities for managing diabetes, yet also reveals unique challenges, particularly in addressing barriers related to digital literacy and the service expectations of patients.

One significant barrier identified in this study is the digital literacy gap among older diabetic patients, which limits their effective engagement with telemedicine. Older adults, who often have greater healthcare needs due to complex conditions such as diabetes, frequently lack the digital skills necessary to navigate online health services, thus impeding their ability to fully benefit from telemedicine (36). Studies consistently emphasize that lower comfort levels with technology among older adults create a substantial barrier to the adoption of telehealth, particularly for those managing chronic conditions that require frequent, consistent interaction with healthcare providers (42, 43). This gap is further compounded for older adults without family support, who may lack immediate assistance in navigating telemedicine platforms (44).

While generic digital literacy programs are commonly implemented, they often fail to meet the unique needs of older adults, especially those in rural or underserved communities where digital support resources are limited (44). Tailored interventions that consider the specific challenges faced by older diabetic patients are therefore essential. Support programs, such as community-based initiatives in partnership with local organizations and NGOs to provide tailored digital literacy support specifically designed for older adults, should be considered for their proven effectiveness in enhancing engagement and adoption (45). This community-driven, tailored approach also demonstrates advantages in reducing anxiety and enhancing self-efficacy, which further improves engagement and satisfaction among older adults in using telemedicine services (46).

Expectations for a seamless, integrated telemedicine service are frequently mentioned as a shared requirement for both providers and users under telemedicine settings within local primary care. The current service model often requires diabetic patients to visit clinics to collect medications following telemedicine consultations, which diminishes the convenience and continuity that telemedicine is designed to offer (47). Studies support the argument that patient satisfaction with telemedicine hinges on the ability to provide a truly remote, uninterrupted healthcare experience, which is particularly valuable for those managing chronic diseases (46, 48). For patients with mobility limitations, this model disrupts their healthcare experience, as they expect telemedicine to deliver a complete, end-to-end solution without in-person clinic visits, which is precisely the purpose that telemedicine is designed to fulfill.

Expanding additional end-to-end services, such as medication delivery, could enable telemedicine to function more independently from in-person clinic visits by removing logistical barriers and aligning services with patient expectations (49). This integration not only enhances convenience but also plays a crucial role in improving patient adherence to treatment. This is essential for managing chronic conditions like diabetes, where consistent access to medications and ongoing monitoring are vital to prevent complications and maintain health outcomes (43, 50). Research has shown that patients are more likely to adhere to medication schedules when medications are delivered directly to their homes, as this eliminates additional trips to healthcare facilities and ensures continuity of care (48, 51).

To ensure the sustainability and effectiveness of telemedicine for diabetes management in local primary care settings, a multifaceted approach is essential. Beyond addressing immediate barriers such as digital literacy and service continuity, long-term success will require ongoing investments in patient-centered infrastructure and support systems. By prioritizing these areas, healthcare providers can create a robust telemedicine framework that not only meets current patient needs but also adapts to evolving demands, ultimately supporting better adherence, engagement, and health outcomes in chronic disease management.

### Strengths and limitations

This study has several strengths. First, the inclusion of a diverse sample of healthcare professionals, encompassing four occupations, ranging from senior medical officers overseeing clinic operations to registration desk staff, enriched data breadth and provided a multi-level view of real-world workflows, barriers, and facilitators. This heterogeneity enhanced the comprehensiveness and practical utility of the findings. Second, the inquiry was embedded in an active Telemedicine Consultation Program within routine primary care, yielding contextually grounded insights that reflect everyday decision-making rather than hypothetical scenarios. This real-world setting strengthens dependability and supports transferability to similar publicly funded primary-care contexts (34, 52). Third, the dual-step analytic approach combined empirical, inductive coding with theory-informed interpretation, allowing us to link emergent themes to established implementation constructs and to generate practice-relevant recommendations. Fourth, we used strategies that bolster qualitative rigor, including investigator triangulation, an audit trail, and thick description of the organizational context and service model, which together enhance transparency and trustworthiness.

This study nevertheless has certain limitations. First, perspectives were limited to healthcare providers; patient and caregiver viewpoints were not captured, which may omit experiential barriers to telemedicine use and equity-related concerns. Future work should include patient and caregiver voices to obtain a more complete assessment. Second, the study was conducted within a single public health system in Hong Kong, which may constrain external validity beyond similar urban, publicly funded primary-care settings. Contextual factors such as infrastructure, digital readiness, and organizational policies could differ in other regions (52). Third, as participation was voluntary, selection bias cannot be excluded; individuals more engaged with telemedicine may have been over-represented. Fourth, social desirability and role-related dynamics in interviews may have influenced responses despite measures taken to ensure confidentiality. Fifth, we did not directly assess implementation outcomes such as cost, time-efficiency, or patient-reported experience, limiting our ability to compare telemedicine with onsite care across economic and experiential dimensions. Finally, using two frameworks may introduce conceptual overlap; we mitigated this by pre-specifying roles for each framework (NASSS for domain identification, ecological lens for level, cross-level links, and temporal dynamics) and by reporting the analytic sequence transparently.

### How this study enhances future research

The themes identified here can directly inform the design and evaluation of future studies. First, they specify patient- and provider-facing determinants that should be targeted in patient-inclusive qualitative work and co-design studies. Second, our context mapping and workflow descriptions can guide multi-site replication studies that test generalizability across different primary-care systems. Third, the implementation barriers and enablers we identify can be translated into measurable constructs and fidelity indicators for hybrid effectiveness-implementation trials, including prospective assessment of cost, digital readiness, and equity impacts. Together, these extensions build a coherent pathway from qualitative insight to testable interventions and policy-relevant evaluation.

## Conclusion

This study identifies key facilitators and barriers to integrating telemedicine into local primary healthcare settings for diabetes management. Using the NASSS framework and Ecological Systems Theory, various factors across multiple levels and stakeholders were examined, providing insights to inform more targeted and effective responses in policy and healthcare practices. Realizing the full potential of telemedicine in primary care requires ongoing efforts to address these challenges and continuously strengthen enabling factors. Future research and practical initiatives should leverage these findings through collaborative efforts among stakeholders to refine the current model, so as to ensure a more effective and sustainable integration of telemedicine into routine healthcare practices.

## Data Availability

The raw data supporting the conclusions of this article will be made available by the authors, without undue reservation.
